# Recent Advances in the Assessment of Cereal and Cereal-Based Product Quality

**DOI:** 10.3390/foods14071220

**Published:** 2025-03-31

**Authors:** Gamze Yazar, Jozef L. Kokini

**Affiliations:** 1 Department of Animal, Veterinary and Food Sciences, University of Idaho, 875 Perimeter Dr., Moscow, ID 83844, USA; 2 Food Science Department, Purdue University, 745 Agriculture Mall Dr., West Lafayette, IN 47907, USA; jkokini@purdue.edu

Cereals are rich in nutrients, such as carbohydrates, fats, proteins, vitamins, and minerals, which make them a very important source of food for the human diet and human health [1,2]. Cereals are typically milled into flours with the goal of mixing them with water and other ingredients to obtain end products with specific quality attributes for the manufacture of bakery and extruded cereal-based products. They are also used as animal feed and human food for enriching the fiber content of foods [3]. The quality of these products greatly depends on the quality and the processing of cereal grains [2]. Therefore, quality assessment of cereal grains and their end-products is an important aspect of the processing and safety of cereal foods [1,4].

Traditional methods used for cereal quality assessment involve wet chemistry methods, high-performance liquid chromatography, enzyme-linked immunosorbent assays, gas chromatography, etc. [1,2]. Even though, these traditional cereal testing methods have been widely accepted and found to be useful by industry to predict end-product quality, there are some limitations associated with them. For instance, they are time- and labor-intensive, often destructive, costly, highly empirical, and non-food-grade chemical-dependent, making them often toxic and harmful to analysts and the environment [1,2,5]. Therefore, recent novel techniques have been used in the cereal science world for the assessment of the quality of cereals and cereal-based products. These techniques include fundamental rheology [6,7,8], bioinformatic modeling [9,10], imaging techniques (i.e., x-ray microtomography [11,12,13], magnetic resonance imaging (MRI) [14,15]), spectroscopy techniques (i.e., near infrared (NIR) [2,16,17], FTIR [18,19,20,21], Raman [17,22,23], nuclear magnetic resonance (NMR) [24,25,26]), 3-D printing technology [27,28] and more.

Physical properties of cereal grains are among the most important parameters defining grain quality. Physically damaged kernels during mechanical harvesting and in the subsequent handling operations make cereals more susceptible to damage from fungi and insects [29]. A significant amount of mechanical damage in kernels leads to a lower grade product with lower market value [30]. Fan et al. [31] used machine vision and machine learning algorithms to develop methods of online rapid detection of the broken corn kernel rate.

Composition of cereals and in particular protein content is known to have a significant effect on end-product quality [32,33,34]. Fan et al. [35] used NIR spectroscopy combined with machine learning algorithms to predict the protein content and its impact on corn kernel quality. Mefleh et al. [36] studied the impact of wheat clipping on the nitrogen content and protein composition in ancient wheats grown in the Mediterranean region. The clipping technique was used to modify grain protein fractions under varying climate conditions. Therefore, it was suggested as a tool to improve the technological quality of grain, rheological properties of the resulting dough, and end-product quality.

Characterization of the rheological properties of cereal flour doughs enables prediction of both the quality of the raw material and the textural characteristics of the end-product [8,33,34]. In the cereal literature, the rheological properties of doughs are commonly determined through empirical methods. Although empirical methods are useful in industrial applications, they provide data in arbitrary units, leading to a difficulty in fundamental interpretation of the results [37]. Yazar [38] reviewed the application of fundamental non-linear rheological tests to offer a more accurate tool to determine the processing quality of wheat flours.

Other studies in this special issue focused on the utilization of novel techniques to improve the nutritional quality of the cereal-based foods. Increasing the dietary fiber intake is globally a key strategy to improve consumer health. For this purpose, the consumption of high-fiber foods, mainly whole grain products, has been promoted. However, whole grain foods have lower acceptability compared to products made from white flour [39]. Sempio et al. [40] used Surface Response Methodology (RSM) as a statistical tool to increase the dietary fiber content of bread. The RSM technique helped optimizing a fiber mixture that could improve the dietary fiber content of bread without altering the desired quality attributes of white wheat bread.

Another global issue for the food industry is to develop new pathways to meet the rising food demand against the continuous increase in the global population and the challenges for environmental sustainability [41,42]. Herdeiro et al. [43] investigated the possibility to develop healthy cereal-based snacks with added edible insects as an alternative protein source. In this study, they used the 3D printing technology to explore its potential in designing novel foods.

The studies in this special issue provide an overall insight into the applications of novel techniques to improve the quality of cereals and cereal-based traditional and future foods.

## Data Availability

No new data were created or analyzed in this study. Data sharing is not applicable to this article.
